# Assessing Shifts in Research Priority Areas From the Neurological Community Before and After the COVID‐19 Pandemic

**DOI:** 10.1111/hex.70504

**Published:** 2026-06-07

**Authors:** Leah Dempsey, Travis Cruickshank, Mitchell Turner, Manja Laws, Danielle Bartlett, Barby Singer, Onno van der Groen, Moira Sim, Mandy Stanley

**Affiliations:** ^1^ Centre for Precision Health Edith Cowan University Joondalup Western Australia Australia; ^2^ School of Medical and Health Sciences Edith Cowan University Perth Western Australia Australia; ^3^ Perron Institute for Neurological and Translation Research Nedlands Western Australia Australia

**Keywords:** consumers, neurological conditions, qualitative, research priorities

## Abstract

**Introduction:**

People living with a neurological condition face many difficulties in daily life, impacting their function and quality of life (QoL). There is currently no cure to many neurological conditions, therefore identifying interventions to improve QoL is of high importance. COVID‐19 changed society in many ways and understanding the research priorities from the neurological community post pandemic is urgently needed to ensure resources are used efficiently and aligned with the needs and priorities of the community. To better understand the priority areas, it is essential for individuals with lived experience to have input into priority areas for research. Therefore, the aim of this study was to identify the top research priorities for the neurological community in Australia.

**Methods:**

This priority setting study had two phases. The first phase comprised a face‐to‐face full day workshop held in late 2019 where participants were led through rounds of brainstorming, categorising and prioritising to reach consensus on a set of research priorities. The second phase was conducted in 2023 with an online survey distributed widely to gauge whether the initial set of research priorities had changed following the event of the global pandemic.

**Results:**

On completion of the 2019 workshop there were a total of 27 priority areas with the top priority being diagnosis and early intervention. The 2023 survey results saw mental health and wellbeing moving up one position to become the highest priority.

**Conclusion:**

Mental health and wellbeing moving from second in 2019 to first in 2023, shows a need for more resources and research into this area for the neurological community. Many participants suggested that mental health is at the centre of their condition and when their mental health is poor it impacts all areas of their life. The research priorities identified in this study provide direction for researchers about what is important to people living with a range of neurological conditions, allowing researchers to focus on the needs of this community.

**Patient or Public Contribution:**

The data collection phase was planned in collaboration with the Consumer and Community Health Research Network, a consumer advocacy organisation. We partnered with people living with neurological conditions for the data collection for both phases and they gave feedback on the findings.

## 1 Introduction

Chronic neurological conditions such as multiple sclerosis (MS), stroke, migraine and Parkinson's disease (PD) have a significant societal and economic impact on the Australian community, with neurological disorders in Australia affecting an estimated 10.6 million people, or just over 40% of the population [1]. It has been estimated that these conditions cost the Australian healthcare system $30.5 billion annually [1]. This underscores the importance of understanding the needs from the neurological community in Australia and developing targeted therapeutic strategies to improve the quality of life (QoL) and to reduce the economic burden of care.

People living with a neurological condition can experience a wide range of problems including, but not limited to, cognitive, sensory and motor impairment, low mood, sleep disturbances, cardiovascular and sexual health problems [2]. These problems may worsen over time, resulting in progressive disability, and associated loss of independence and, in some cases, increased morbidity. The progressive loss of function as well as the uncertainty about the likelihood of progression and rate of deterioration in some neurological conditions can severely impact the QoL of these individuals and their families. While it is important to target all areas of health, there needs to be an understanding of what is most important to the individual, and what therapy may provide an increase of QoL.

During the COVID‐19 pandemic, people living with a disability were more likely to report negative financial impacts compared to individuals without a disability [3]. The impact was also felt in access to health care, during times of lockdown individuals living with a neurological condition reported disruptions to treatment, including delayed therapies and inability to access treatment from pharmacies [4]. These consequences from lockdown and the pandemic have seen a negative impact on mental health and increased anxiety and loneliness on individuals with a neurological condition and other disabilities [3, 4].

Despite intense scientific efforts, there is no cure or disease‐modifying therapies for many chronic neurological conditions. Medications to treat symptoms are also accompanied by side effects, leading individuals to seek nonpharmacological therapies [5]. Over the last two decades, substantive evidence has emerged of the benefits of non‐pharmaceutical approaches, such as physical activity programs, mental health and mindfulness training and cognitive behavioural therapy, for the management of symptoms and, in some cases, slowing of disease progression in individuals with these conditions [6, 7, 8]. However, due to the COVID‐19 pandemic publishing rates of research unrelated to COVID‐19 pandemic has experienced a significant drop and many therapeutic trials were postponed [9]. This may have significant consequences for the neurological community as it may delay access to new and improved treatments [9]. While the research areas unrelated to COVID‐19 are recovering post pandemic there is an urgent need to identify the research priority areas for the neurological community to avoid any further reduction in research output for diseases that do not fall under COVID‐19 related research.

Engagement with consumers is recognised as a critical step in developing health research projects. It is a way for the affected members of the community to contribute knowledge based on their lived experience, in keeping with the well‐known phrase ‘Nothing about me without me’ [10]. Involving those who will directly benefit from the research findings in the priority‐setting process increases the impact of research, as well as the likelihood that its outcomes will be adopted in clinical practice [11]. There have been research priority setting exercises conducted with those with lived experience in a range of health conditions, including stroke, PD, chronic fatigue syndrome/myalgic encephalomyelitis, asthma, cancer and mental health [12, 13]. To date, we have not found documentation of the inclusion of consumers living with a broad range of neurological conditions in a priority‐setting exercise conducted in Australia, yet there are commonalities in areas of priority across a range of neurological conditions. Therefore, the aim of this study was to identify the top research priorities for the neurological community in Australia. At the time of starting this exploration there was little knowledge about what the impact would be on the health and well‐being of people world‐wide in a global pandemic. We therefore sought to confirm that the priorities identified in phase 1 of this study were still relevant for the target population post pandemic.

## 2 Materials and Methods

### 2.1 Priority Setting Workshop 2019

To enhance a major program of research (Systematic Profiling in Neurological Conditions [SPIN]), the research team engaged with people with lived experience of a range of neurological conditions to ascertain their perspective on priorities in research. Prioritising the views of those with lived experience was important for a range of reasons: we wanted our research to be relevant and useful, and we were aware that recruitment for research studies would be more successful if we were pursuing areas that were a priority for the community. The first phase of this study was conducted in 2019 and involved a workshop with people with lived experience and other stakeholders in Perth, Western Australia (WA). To address the research question, a consensus building approach together with a modified nominal group technique was used [14]. Working in collaboration with the Consumer and Community Health Research Network and our funding partner the Multiple Sclerosis Society of Western Australia (MSWA), we invited people living with a neurological condition and their carers to participate in a half‐day workshop to discuss their views on what should be considered as the research priority areas for the SPIN research program. Ethical approval for the study was provided by the Human Research Ethics Committee, Edith Cowan University (Approval number 2019‐006060‐SIM) and participants were provided with an honorarium in recognition of their time. Before the workshop, potential participants were provided with an information sheet and consent form and the questions that were to be covered during the workshop to allow sufficient time to consider their views and to think about what they would like to contribute to the discussion. The questions were as follows:

1. What do you believe are the main issues impacting on the lives of people living with a neurological condition?
2. Which issues would you like to see addressed in future research for individuals living with a neurological condition?

The workshop took place over 5 h, and morning tea and lunch breaks were included. Research staff took the opportunity for one‐to‐one conversations with participants during the breaks. Upon arriving at the workshop, which was held at an accessible venue, data via a short questionnaire were collected about age, gender, postcode (to determine metropolitan, rural and remote attendees) and identification as either an individual with lived experience of a neurological condition or as a carer.

The workshop was jointly facilitated by a group of facilitators with extensive experience in consulting with consumers, including the senior author (M.S.). The workshop began by briefly describing the background of the study and the purpose of the workshop to give consumers some context to the discussion. Using a combination of small group work and nominal group technique, participants were led through a series of activities to develop a list of research priorities. Participants were seated at tables with a facilitator and a note‐taker at each table. The first activity consisted of brainstorming and discussion of the two abovementioned research questions and the generation of a list of potential research areas. Over the refreshment break, the facilitators collated the ideas from each table and, through a rapid process of discussion, generated common themes that accounted for all the contributed ideas.

The second activity was a discussion at each table of the themes that were generated from the first activity. Each table worked through a process to arrive at consensus of the ranking of priority of the theme. Over the next refreshment break, the rankings for each table were combined to arrive at a set of ranked priorities for the whole workshop group. These were discussed as a whole group so that participants could clarify meaning if required and debate rankings. By the end of the workshop, a list of research priority areas, ranked according to level of importance for workshop attendees, had been generated to guide subsequent research project development. Data from the workshop included notes taken by research assistant observers, notes collated at each table and records of the outcomes of the nominal group techniques. The notes from the workshop provided an audit trail of the process and informed the development of subsequent presentations and publications. The generated list of priorities was entered into an online survey in Qualtrics to distribute to individuals living with a neurological condition who were not able to attend the workshop in person. The aim was to give those living in rural or remote areas of WA, or who have difficulties with community mobility, to add their perspective. The link to the survey was distributed widely using social media and in newsletters.

### 2.2 Priority Setting Survey 2023

To determine if the ranked priority areas developed during the 2019 workshop described above were still relevant to the neurological community post‐COVID‐19 pandemic, an online priority‐setting survey was developed. Ethical approval for the study was provided by the Human Research Ethics Committee, Edith Cowan University (approval number 2023‐04609‐DEMPSEY). The survey was built on the priority research areas developed during the earlier workshop. The list of priority areas developed during the 2019 workshop were entered into an online survey on Qualtrics, along with some additional questions addressing people's willingness to participate in further research. After obtaining consent, demographic data were collected, including age, postcode, and whether they identified as an individual with lived experience of having a neurological condition or as a stakeholder, including carers, family members, healthcare professionals and community members. The survey then displayed the list of 27 priority areas identified in the 2019 workshop, detailed in Section 3 below, and participants were asked to rank these areas in order from most important to least important. An additional open text box response was added, where participants could include areas that may have not been listed.

The survey was made public from October 2023 until mid‐December 2023. The survey was distributed through the SPIN Registry database, which includes people living with a range of neurological conditions, carers, family members of those living with a neurological condition and health professionals. Further, the survey was disseminated on social media, including Facebook, the Stroke Foundations Enable Me platform and industry newsletters such as the Stroke Foundation newsletter.

### 2.3 Statistical Analysis

Data from the 2023 online survey were exported from Qualtrics into MS‐Excel and analysed quantitatively. The order of the ranking was calculated using the mean of each priority area in ascending order.

## 3 Results

### 3.1 Results From the 2019 Workshop

In all, 22 consumers participated in the workshop, including 3 carers and 19 individuals living with one or more neurological conditions. Most individuals reported living with MS (12), while 2 individuals were living with Motor Neurone Disease and 5 individuals were living with other neurological conditions (including but not limited to PD). Most of the participants were older than 50 (13), with three participants reporting to be in their 30s. A total of 11 males and 8 females living with neurological conditions participated in the workshop. All carers were female.

Table 1 lists the initial unranked priority areas generated after the first round of collating information from each table.

**Table 1:** Collated list of priority areas for research.

A. Emotional well‐being and mental health	N. Cure
B. Sleep, tiredness, exhaustion, fatigue	O. Funding
C. Access to support (financial, physical)	P. Consumer involvement in research
D. Pain and other sensory issues	Q. Longitudinal research
E. Quality of life (fun, sex life and lifestyle)	R. Complimentary therapies
F. Fear of disease progression	S. Side effects of drugs
G. Diagnosis, early intervention	T. Trauma
H. Lack of understanding, education of medical practitioners	U. Use of technology
I. Limited treatment options	V. Holistic approach
J. Mobility issues (balance, strength)	W. Collaborative cross‐disciplinary research
K. Impact of environment	X. Risk factors
L. Management of condition (self, team)	Y. Communication of research findings
M. Stigma and awareness of community	Z. Nutrition, gut microbiome
	ZZ. Carers

The priority areas from each table were collated into one set of rankings. Though the process involved consensus building, the final list of agreed priorities was as presented in Table 2.

**Table 2:** Final research priorities (2019).

1. Diagnosis/early intervention
2. Mental health and emotional well‐being
3. Cure
4. Pain and other sensory issues
5. Sleep, tiredness, exhaustion and fatigue
6. Management of condition
7. Lack of understanding and education of medical practitioner and wider community
8. Funding
9. Gut health, nutrition and microbiome
10. Limited treatment options
11. Mobility issues
12. Quality of life
13. Access to support
14. Risk factors
15. Fear of disease progression
16. Carers
17. Side effects of drugs
18. Consumer involvement in research
19. Impact of the environment
20. Holistic approach
21. Stigma and awareness of community
22. Complimentary therapies
23. Collaborative, cross‐disciplinary research approach
24. Use of technology
25. Trauma
26. Longitudinal research
27. Communication of research findings

A summary of the findings was distributed to the participants inviting further feedback. Several participants took up that opportunity to express thanks for inviting them to be involved which we took as confirmation and validation of the outcome.

### 3.2 Results From 2023 Online Survey

The survey was completed by 139 individuals; the demographic breakdown can be seen in Table 3. The most common types of neurological conditions included MS (15), functional neurological disorder (8) and PD (6).

**Table 3:** Demographics for 2023 survey.

Participants	
Individuals living with a neurological condition	110
Other stakeholders	29
**State or Territory**	
Western Australia	77
New South Wales	21
Australian Capital Territory	2
Victoria	13
South Australia	8
Tasmania	1
Northern Territory	2
Queensland	15

Ranking of the priority areas from the 2023 Qualtrics survey is presented in Table 4. A sub‐analysis was completed on the WA population only; however, this did not significantly change the ranking of the priority areas.

**Table 4:** Priority rankings 2023 (*N* = 139).

	Field	Mean SD	Mode
1	Emotional well‐being and mental health	7.25 ± 6.62	2
2	Sleep, tiredness, exhaustion, fatigue	8.42 ± 8.07	2
3	Quality of life (fun, sex life, lifestyle)	9.86 ± 7.18	6
4	Diagnosis, early intervention	9.91 ± 7.33	1
5	Management of condition (self, team)	10.09 ± 5.94	13
6	Pain and other sensory issues	10.40 ± 7.53	2
7	Lack of understanding, education of medical practitioners	11.22 ± 6.89	8
8	Access to support (financial, physical)	11.68 ± 7.35	8
9	Mobility issues (balance, strength)	11.94 ± 6.89	4
10	Cure	12.88 ± 8.29	1
11	Limited treatment options	12.92 ± 6.69	10
12	Fear of disease progression	13.41 ± 8.41	25
13	Side effects of drugs	13.76 ± 6.92	7
14	Stigma and awareness of community	14.77 ± 6.24	14
15	Holistic approach	15.23 ± 7.25	22
16	Funding	15.52 ± 5.97	17
17	Consumer involvement in research	15.95 ± 6.38	18
18	Complementary therapies	15.98 ± 6.37	17
19	Collaborative cross‐disciplinary research	16.35 ± 7.38	23
20	Communication of research findings	16.80 ± 7.64	25
21	Longitudinal research	16.85 ± 6.49	19
22	Trauma	16.92 ± 7.24	20
23	Nutrition, gut microbiome	17.82 ± 7.75	26
24	Risk factors	18.31 ± 7.14	24
25	Use of technology	18.65 ± 6.24	24
26	Impact of environment	18.91 ± 5.90	27
27	Carers	19.42 ± 7.61	27
28	Other	24.78 ± 7.93	28

The top 10 priorities are elaborated below.

1. Emotional well‐being and mental health
  Participants were interested in understanding how they can maintain or improve their mental health and well‐being in the context of living with a neurological condition that might be unpredictable and progressive. ‘If emotionally and mentally unwell and in pain, quality of life will be poor no matter what else is done’, was articulated by one of the participants living with a neurological condition. A participant who identified as a carer of someone living with a neurological condition stated, ‘The young person I support is often extremely anxious and depressed … We can't find a suitably qualified or experienced psychologist ‐ they all have long waiting lists’.
2. Sleep, tiredness, exhaustion and fatigue
  Fatigue and poor sleep health have been identified as a problem area across multiple neurological conditions [15, 16, 17]. This issue moved from the fifth priority in 2019 to second in 2023. One participant commented, ‘This has a huge potential impact on everything that I do. Some days (often) I feel like I'm getting on top of things and am looking positively at getting things done and achieving them throughout my day and then the fatigue just hits me and takes over and there's nothing I can do about it’.
3. QoL (fun, sex life and lifestyle)
  QoL moved up the priority list significantly from twelfth in 2019 to third in 2023. As is evident in the following quotes from participants living with neurological conditions participants choosing this priority area as their top priority did so because of its impact on treatment and disease outcomes. ‘This year I have learnt/realised how much fatigue, and quality of life is affected by MS and it was VERY scary! ensuring patients have quality of life is critical to all ethics’, and ‘…without living a reasonable quality of life, there is little point in treatment’.
4. Diagnosis, early intervention
  Diagnosis and early intervention remained high on the priority list. Some participants shared their personal struggles of achieving a diagnosis in the early stages of their condition while others stated that research needs to be done in this area to achieve the best outcomes for future generations. One participant shared their reasoning, ‘Diagnosis & early intervention can mean harm minimisation. It can mean better outcomes in the long run…. This area is a priority for more research as we can then know what techniques, pharmacology etc should be employed as early as possible in order to get the best outcomes’. Having a diagnosis validates the person's experience and enables them to seek appropriate treatment and adapt to life with a neurological condition.
5. Management of condition (self, team)
  Any research that advances knowledge of best practice in managing neurological conditions is seen as desirable. The 2023 survey sample included participants with rare neurological conditions, who expressed their concern about the limited treatment options and lack of intervention available. One individual living with Idiopathic intracranial hypertension stated, ‘The treatment options available are so limited that management of the condition is difficult’. Another expressed their desire for medical health professionals to work together in a team approach, ‘It's difficult for all the various doctors and allied health practitioners to collaborate and plan care properly. I would love to see a facility where these health professionals work together to treat neurological conditions’.
6. Pain and other sensory issues
  Participants sought answers to how they might be able to better manage issues such as pain, and problems with sensation, vision and hearing. The interference of pain and other sensory issues on participation detracted from QoL, impacting on their ability to engage in a full life.
7. Lack of understanding, education of medical practitioners
  Individuals living with a neurological condition often talk about their health professionals having a lack of understanding about their condition and their lived experiences. As one participant stated, ‘Being heard helps with healing’. These concerns are common amongst those living with rare conditions that may be under‐researched. A participant living with Myalgic Encephalomyelitis expressed their concern on the lack of understanding and research on the condition. Stating ‘My disease is a much neglected area of research with only a limited number of researchers actively involved. … I have had to do my own research due to a lack of interest and other patients do too’.
8. Access to support (financial, physical)
  Access to support is often identified as a priority area for this population within Australia. Location may be an issue with individuals having to travel from rural or remote areas to receive treatment or attend medical appointments. One participant stated there is ‘…not enough support provided by society’. A comment was made on the disparity in costs of services between metropolitan and regional areas, with the example used of a cost of a therapeutic massage being significantly more in Broome compared to Perth.
9. Mobility issues (balance, strength)
  Mobility issues are commonly recognised as a priority area for the neurological community as many conditions cause physical impairment. As one participant stated, ‘Mobility issues suck!’. Poor mobility was linked to poor mental health due to restrictions on freedom of movement and poor community access for socialising and other activities. Research has increased in addressing this area of concern, however more support is needed within the population to address their needs [6].
10. Cure
  It was acknowledged that people living with a neurological condition make the best of their life, but most people would rather not have to live with the symptoms, the burden of day‐to‐day management and the uncertainty about the future.
  ‘Cure from PD is my top priority. Whilst it will not affect me it is possible that my children and grandchildren may be susceptible to the disease. To be cured at a young age or better still to be provided with a preventer would remove their fear and mine for the future. This surely has to be the highest priority’.

## 4 Discussion

The aim of this study was to gain an understanding of the issues facing individuals living with neurological conditions and to determine the priority research areas that should be targeted in future research to improve their QoL. The workshop and subsequent survey provided an opportunity for individuals living with neurological conditions and relevant stakeholders to have an input into future research directions. All data collection methods were well received by participants who expressed appreciation that they were consulted and that their perspective was valued. Following the event of the global pandemic it was important to explore if the prior research priorities collected in the first phase of this study still held.

The first phase of data collection for this study occurred in late 2019, just before the global pandemic becoming evident in Australia. The follow up data collection by online survey showed that the priorities had changed with mental health now being the top priority. This is perhaps not surprising given the overall increase in anxiety associated with the pandemic which has been well documented [18]. Those living with a neurological condition potentially experienced an increased level of vulnerability to the COVID‐19 virus and the potential consequences given their pre‐existing condition and greater disruption to the ability to manage their own condition. The findings highlight the need for holistic approaches that address the whole person, not just a focus on management of physical symptoms associated with neurological conditions. Participants expressed the desire to be supported, engaged and empowered to participate in their own self‐management.

Emotional well‐being and mental health were considered a top research priority in the neurological community in Australia, sitting as the second highest priority in 2019, then moving to the top priority in the 2023 survey. Participants stated that mental health and well‐being is at the centre of their condition, suggesting that if their mental health is poor all other areas of their life will suffer. Living with a neurological condition is often associated with higher levels of stress, a perceived lack of control and feelings of helplessness and anxiety [19]. The presence of depression and anxiety has been shown to have correlations with disease symptoms and disease management, resulting in significant fatigue, pain and sleep problems, lowering overall QoL and increasing carer burden [19, 20]. Increased rates of depression are likely to be associated with increased rates of suicide, indeed suicide risk in the MS population is twice that in the general population [21]. These findings speak to the importance of identifying mental health illness in neurological populations and providing adequate treatment and interventions to support these concerns.

Some of the findings of this study are similar to previous priority setting exercises focussed on a single neurological condition. For example, sleep and fatigue rated sixth out of the top 10 priorities for people following stroke and eighth for people with PD [22, 23]. Sleep, tiredness, exhaustion, fatigue ranked as the 2nd highest priority in the 2023 survey, confirming that this area is still of high importance and has become more important than previously reported. It is well documented that people living with MS, PD or post stroke suffer from issues with sleep and fatigue, leading to an impact on participation and thus QoL and well‐being [24, 25, 26, 27]. Sleep disturbances have been linked to diminished visual memory, verbal memory and executive function in individuals living with MS [28, 29]. Emerging research has shown promising results to address sleep and fatigue in MS through occupational therapy interventions using sleep hygiene, CBT, environmental modification and an exercise intervention post‐stroke [30, 31]. However further robust studies with well‐articulated intervention protocols are warranted. There is an urgent need for studies to address this issue, as improving sleep quality and reducing fatigue is likely to provide significant improvements to the lives for those affected.

Diagnosis and early intervention were the top priority in the 2019 workshop; however, this area dropped to fourth in 2023. One reason for this may be that the workshop only included individuals from Western Australia, whereas the 2023 survey was disseminated nationally. Regardless of the reduction in priority, receiving a diagnosis, even if it confirms people's fears, validates their experience and enables them to seek appropriate management including education and support [32].

The neurological community who was involved in this study wanted to be a part of the solution and use their lived experience to be involved in research studies. Being a research participant enables individuals to have a sense of contributing to society and having a sense of purpose [33]. There are also numerous calls in the literature and from the participants in this study to communicate research findings in multiple forms and in ways that can be understood by a nonacademic audience. As identified by Lacerda et al. [33] if findings are linked to compelling real stories or to celebrities, they are more likely to attract media attention resulting in broader dissemination of results than if findings are limited to academic publications. Researchers are strongly encouraged to consider research studies that involve partnering with those with lived experience and the use of co‐design as well as publishing lay summaries. Besides this, researchers are encouraged to focus early on in the research design on how research might translate into practice.

## 5 Limitations

The chosen data collection approach did not allow for inclusion of the perspectives of people for whom English was not their first language, or who had speech and/or language or significant cognitive difficulties. There is a need for the development and inclusion of methods of data collection within priority setting exercises that enable wider participation to account for diversity including funding for the use of interpreters or for working with individuals with communication difficulties. It is possible that the perspectives presented here do not speak to priorities for people from more culturally diverse backgrounds. There was a small sample size for the workshop and attendance was limited to those who lived within the metropolitan area of Perth, had transport and were able to attend on the day. However, sample sizes in qualitative research are not intended to be representative and the richness of data outweighs the size of the sample. There were limitations with the survey, due to the size of the priority area lists it was more easily completed on a laptop or computer, this may have excluded individuals without these devices. The survey was available for a short period of time, this reduced our sample size and reach. Findings may be interpreted with some caution given the unique context of Western Australia but it is likely that findings would be similar in other contexts.

## 6 Conclusion

The findings of this two‐phased study have provided an insight into the priorities of the neurological community in Australia, providing guidance to researchers on what is important for people living with neurological conditions and where those with lived experience would prefer research efforts to be directed. Knowledge of the priorities will enable researchers to strengthen the rationale and justification for their research studies within grant applications and ethics protocols. If researchers' efforts are matched and targeted towards the priorities of those with lived experience it is likely to enhance recruitment leading to greater statistical power, and greater uptake of findings. More importantly if research efforts are directed to what matters to people living with neurological conditions it should empower them to live their best life.

## Author Contributions

**Leah Dempsey:** writing – original draft, methodology, investigation, formal analysis, software. **Travis Cruickshank:** investigation, funding acquisition, writing – original draft, writing – review and editing, supervision. **Mitchell Turner:** investigation, writing – original draft, writing – review and editing, formal analysis, supervision, software. **Manja Laws:** project administration, writing – review and editing, funding acquisition, supervision. **Danielle Bartlett:** writing – review and editing. **Barby Singer:** writing – review and editing, investigation. **Onno van der Groen:** writing – review and editing. **Moira Sim:** writing – review and editing, funding acquisition, investigation. **Mandy Stanley:** conceptualisation, writing – original draft, methodology, writing – review and editing, formal analysis, supervision.

## Ethics Statement

This study was approved by the Edith Cowan University Human Research Ethics Committee (2019‐00970‐LAWS) (2019‐006060‐SIM) (2023‐04609‐DEMPSEY).

## Consent

Testing procedures were conducted in accordance with the Declaration of Helsinki and written informed consent was captured before study commencement.

## Conflicts of Interest

The authors declare no conflicts of interest.

## Supporting information

Priority_Setting_Survey.

## Data Availability

Data sharing not applicable to this article as no datasets were generated or analysed during the current study.
