# Supplementary material for: Assessing Shifts in Research Priority Areas From the Neurological Community Before and After the COVID‐19 Pandemic
Source: Health Expect. 2026 Jun 7;29(3):e70504. doi: 10.1111/hex.70504 (PMC13242650; doi:10.1111/hex.70504)
Supplement: Supplementary file 1 — Priority_Setting_Survey. [file HEX-29-e70504-s001.docx]

Priority Setting Survey

Start of Block: Introduction and consent

Q18

Q4 **You are invited to participate in a Research Priority Setting (RPS) Survey which seeks to understand the issues that people living with a neurological condition face in their day-to-day life and the aspects of the conditions that are most important to you. You are being asked to take part in this project to provide you with an opportunity to express your opinions in regard to future directions of research into neurological disorders.** Please read through all the information carefully. If you do not understand or want to know more about specific aspects of the study, please do not hesitate to contact the principal investigator of the study, Ms. Leah Dempsey (contact information is provided at the end of this document). It is worthwhile consulting with a relative or friend prior to participating in this research study. Participation in this research is voluntary. If you do not wish to take part, you do not have to.  If you do decide to take part and later change your mind, you are able to withdraw (quit) at any time. Please note that if you do withdraw, it will not be possible to retract the data you provided during the survey, as no information allowing you to be identified will be collected during the survey to maintain anonymity. If you decide you want to take part in the research project, you will be asked to consent before the beginning of the survey by ticking a checkbox. By consenting to participate, you are indicating to us that you: Understand the information provided in the participant information sheet and consent form Consent to participate in the research project Consent to undertake the assessments and procedures that are described Consent to the use of your personal and health information as described

Q6 Study location This project will be conducted remotely via online survey.   Who is conducting this research? This project is conducted by Systematic Profiling in Neurological Conditions (SPIN) researchers at Edith Cowan University. What is this project about? The aim of this Research Priority Setting Survey is to gain an understanding of the issues currently facing individuals living with a neurological condition and the areas that could be targeted in future research trials to improve day-to-day life. This survey will provide an opportunity for individuals living with neurological conditions, their families, and carers to have an influence on the future research directions. This project is run within the Systematic Profiling in Neurological Conditions (SPIN) Research Program and has been funded by MSWA. What does participation in this research involve? Your participation in this research project will involve the completion of a short (15 minute), once-off online survey. The survey will provide you with a list of priority areas identified during a workshop held by the SPIN team in 2019. We would like you to rank these priority areas from most important to least important. For your top choice, we ask you short additional questions to understand your reasoning. At the end of the survey, we will ask you some questions investigating your motivation to participate in research. By the end of the project, we anticipate having a list of the highest priority areas for research to guide future work. We will also ask a few questions about yourself, such as age, whether you are an individual with a neurological condition, a carer or family member or health professional; year of diagnosis and diagnosis (if applicable). Contribution to the survey does not imply any commitment to further research participation. Only the individuals named as investigators on this research project will have access to the collected data.

Q7 Your privacy By selecting “yes” on the survey consent form, you agree to the research team collecting and using information about your personal views on your health condition for the research project. As no personally identifying information will be collected, your responses during the survey will remain anonymous.  Your information will only be used for the purpose of this research project and it will only be disclosed with your permission, except as required by law. It is anticipated that the results of this survey will be published and/or presented in a variety of forums.  In any publication and/or presentation, information will be provided in such a way that you cannot be identified. All data collected will be stored in an anonymous manner on a secure server for a minimum of seven years. Following this period, all data will be permanently deleted. What are the benefits of participating in this research? This Research Priority Setting survey will not lead to any direct personal benefits. However, by providing information on the most impactful aspects of your condition, you will help to provide a foundation for future research projects that will benefit the neurological community. What are the possible risks and disadvantages of taking part? While the project focuses on research priorities, some people may experience a level of discomfort. Should you experience any discomfort we encourage you to talk to a family member or your treating physician. If this is not an option for you, please find contact details for the following helplines: Lifeline Australia: **13 11 14** Beyond Blue: **1300 22 4636** We will also include these details at the end of the survey should you feel the need to speak to someone.

Q8 Who has reviewed this research project? An independent group of people called a Human Research Ethics Committee (HREC) reviews all research in Australia involving people. The ethical aspects of this research project have been approved by the HREC at Edith Cowan University (study identification number 2023-04609-Dempsey). What if I have complaints? If you suffer any injuries or complications as a result of the research project, you should contact the research team as soon as possible. If you are eligible for Medicare, you can receive any medical treatment required to treat the injury or complication, free of charge, as a public patient in any Australian public hospital. If you would like to speak to an independent party about this research project, you may contact the Edith Cowan University Human Research Ethics Committee (phone: (08) 6304 2170, email: research.ethics@ecu.edu.au). Kindly mention the study identification number (2023-04609-Dempsey) in your communications to help with the processing of your request.   Contacting the research investigators? We are happy to answer any questions you may have now, or at any point throughout the study. Please contact the Principal Investigator with your queries:   Ms. Leah Dempsey Phone: (08) 6304 9821 Email: l.dempsey@ecu.edu.au or spin@ecu.edu.au   If you have concerns about the research and wish to speak to an independent party, you may contact:   Edith Cowan University Human Research Ethics Committee  Phone: (08) 6304 2170  Email: research.ethics@ecu.edu.au           *Thank you for taking the time to read through this information sheet, to participate in this survey please select yes to the question below.*     *Principal Investigator*    Chief Investigator: Ms Leah Dempsey School of Medical and Health Sciences Centre for Precision Health Edith Cowan University 270 Joondalup Drive JOONDALUP WA 6027 Phone: +61 8 6304 Email: l.dempsey@ecu.edu.au To download a copy of the information sheet please click Here

Q3 Do you wish to participate in this research?   (By selecting yes you are indicating that you have read and understood the requirements of the research project.)

- Yes (1)
- No (2)

Skip To: End of Survey If Q3 = No

End of Block: Introduction and consent

Start of Block: Demographic Questions

| 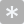 |
| --- |

Q11 Please enter your post code.

________________________________________________________________

| 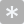 |
| --- |

Q10 Please enter your date of birth. DD/MM/YYYY

________________________________________________________________

Q9 Which of the following best describes you?

- An individual living with a neurological condition (1)
- A carer of someone living with a neurological condition (2)
- A family member or friend of someone living with a neurological condition (3)
- A member of the community (4)
- A health care professional (5)
- Other (6) __________________________________________________

Skip To: Q12 If Q9 = An individual living with a neurological condition

Skip To: Q13 If Q9 = A health care professional

Display this question:

If Q9 = An individual living with a neurological condition

| 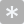 |
| --- |

Q12 Please enter your neurological condition.

________________________________________________________________

Display this question:

If Q9 = An individual living with a neurological condition

Q17 Please enter your year of diagnosis

________________________________________________________________

Display this question:

If Q9 = A health care professional

Q13 Please enter your occupation.

________________________________________________________________

End of Block: Demographic Questions

Start of Block: Priority areas

| 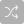 |
| --- |

Q2 **Please rank the list of priority areas from most important (1) to least important (28).** Click and drag your statement to the preferred order.  (If you are completing this survey on your phone, it may be easier to rotate your phone to landscape)

______ Emotional well-being and mental health (1)

______ Sleep, tiredness, exhaustion, fatigue (2)

______ Access to support (financial, physical) (3)

______ Pain and other sensory issues (4)

______ Quality of life (fun, sex life, lifestyle) (5)

______ Fear of disease progression (6)

______ Diagnosis, early intervention (7)

______ Lack of understanding, education of medical practitioners (8)

______ Limited treatment options (9)

______ Mobility issues (balance, strength) (10)

______ Impact of environment (11)

______ Management of condition (self, team) (12)

______ Stigma and awareness of community (13)

______ Cure (14)

______ Funding (15)

______ Consumer involvement in research (16)

______ Longitudinal research (17)

______ Complimentary therapies (18)

______ Side effects of drugs (19)

______ Trauma (20)

______ Use of technology (21)

______ Holistic approach (22)

______ Collaborative cross-disciplinary research (23)

______ Risk factors (24)

______ Communication of research findings (25)

______ Nutrition, gut microbiome (26)

______ Carers (27)

______ Other (28)

Q16 Think about your top priority area. Why is this area of research important to you?  Please tell us more about why this topic is a priority for more research

________________________________________________________________

________________________________________________________________

________________________________________________________________

________________________________________________________________

________________________________________________________________

End of Block: Priority areas

Start of Block: Research

Q15 Would you volunteer in a research program?

- Yes (1)
- No (2)

Q14 Please provide your reasoning for volunteering or not volunteering in a research program.

________________________________________________________________

| 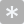 |
| --- |

Q16 What is the maximum trial length that you would be comfortable to participate in? Please provide your answer in weeks.

________________________________________________________________

Q17 What would motivate you to participate in research?

________________________________________________________________

End of Block: Research
